# Network medicine framework identified drug-repurposing opportunities of pharmaco-active compounds of Angelica acutiloba (Siebold & Zucc.) Kitag. for skin aging

**DOI:** 10.18632/aging.204789

**Published:** 2023-06-12

**Authors:** Jiaxin Mo, Zunjiang Li, Hankun Chen, Zhongyu Lu, Banghan Ding, Xiaohong Yuan, Yuan Liu, Wei Zhu

**Affiliations:** 1 The Second Clinical College, Guangzhou University of Chinese Medicine, Guangzhou Province 510006, China; 2 Guangdong Provincial Hospital of Traditional Chinese Medicine, Guangzhou Province 510120, China; 3 Guangzhou Qinglan Biotechnology Co. Ltd., Guangzhou Province 515000, China; 4 Guangzhou Huamiao Biotechnology Research Institute Co. Ltd., Guangzhou Province 510000, China

**Keywords:** network medicine framework, drug-repurposing opportunities, Angelica acutiloba (Siebold & Zucc.) Kitag., skin aging, pharmaco-active compounds

## Abstract

Increasing incidence of skin aging has highlighted the importance of identifying effective drugs with repurposed opportunities for skin aging. We aimed to identify pharmaco-active compounds with drug-repurposing opportunities for skin aging from Angelica acutiloba (Siebold & Zucc.) Kitag. (AAK). The proximity of network medicine framework (NMF) firstly identified 8 key AAK compounds with repurposed opportunities for skin aging, which may exert by regulating 29 differentially expressed genes (DGEs) of skin aging, including 13 up-regulated targets and 16 down-regulated targets. Connectivity MAP (cMAP) analysis revealed 8 key compounds were involved in regulating the process of cell proliferation and apoptosis, mitochondrial energy metabolism and oxidative stress of skin aging. Molecular docking analysis showed that 8 key compounds had a high docked ability with AR, BCHE, HPGD and PI3, which were identified as specific biomarker for the diagnosis of skin aging. Finally, the mechanisms of these key compounds were predicted to be involved in inhibiting autophagy pathway and activating Phospholipase D signaling pathway. In conclusion, this study firstly elucidated the drug-repurposing opportunities of AAK compounds for skin aging, providing a theoretical reference for identifying repurposing drugs from Chinese medicine and new insights for our future research.

## INTRODUCTION

Skin aging, a complex and unavoidable process among the elderly population, has attracted increasing attention around the world. It is mainly charactered by progressive dysfunction and decreased regenerative potentiality of skin layers [1], which appears as laxity, dryness and some facial exaggerated expression lines, xerosis [2]. Nowadays, with a high rate up to 13.7% of aged population in the world (https://population.un.org/), aging has been the principal consideration leading to the prevalence of skin aging and barrier dysfunction. However, another important exterior factor should also be taken into account, that is ultraviolet radiation (UVR) exposure, which mainly includes ultraviolet radiation A (UVA) (90–95%) and ultraviolet radiation B (UVB) (5–10%) [3]. UVR exposure could accelerate skin degradation and photoaging, leading to damage of skin appearance, physiological function, and even melanoma or non-melanoma skin cancer [4]. UVA and UVB exposure are considered to be the potent driver for photodamaged skin induced by oxidative stress injury and free radical damage [5]. The most popular approach in protecting the skin from UVR is the application of shading equipment (umbrellas, hats, long sleeved clothes etc.) and topical application of sunscreens. Although some oral and topical drugs with skin photoprotective effects were recommended, most of products still lack convincing protective functions, which unveiled the need and significance for the development and deployment of new drugs with potential effectiveness for skin aging.

Network medicine framework (NMF) has been applied for unveiling the complex role between drugs and diseases by capturing the molecular interaction between drugs and their targets [6]. The proximity between the targets of compounds and diseases with human interactome is calculated to identify their potential rational drug repurposing [7–9]. It could enhance the confidence and accuracy of predictive power by calculating the discernible adjacent regions between the targets of AAK component and the human interactome, thus the proximity between compound and disease targets could be applied for identified the therapeutic effects of AAK for skin aging. Deisy Morselli Gysi et al. published their results on the Proceedings of the National Academy of Sciences (PNAS) suggesting that the application of NMF could quickly and correctly screen new drugs with repurposing opportunities for COVID-19 [9], which greatly reduce time and economic cost of new drug development in the context of a viral pandemic. The results published on Nature Food also proved that calculating the proximity of NMF between polyphenol targets and disease proteins could precisely predict and screen polyphenols with therapeutic effects [8]. The results of Fang J et al. published in Nature Aging also successfully identified sildenafil with repurposing opportunities for Alzheimer’s disease by NMF [10]. NMF is mainly used to explore the new therapeutical effect of drugs [11], and the rug-repurposing opportunities from one disease to another [12]. Thus, the NMF exerts a significant role in correctly revealing drug-repurposing opportunities for new drugs, especially for nature products, which contain multiple compounds [8, 9, 13].

Traditional Chinese medicine (TCM) has exerted a vital role in skin aging during the past decades, it is reported that TCM has been the main source of new drugs for multiple nature compounds [14]. TCM exerts therapeutic effect on skin aging relying on complex molecular mechanisms of multi-compounds, multi-targets and multi-pathways [15], which conceals the role of single components with significant efficacy. For example, artemisinin, exploited from Artemisia annua L., was proven as an antimalarial drug against drug-resistant plasmodium falciparum [16]. This herb was employed for relieving malaria symptoms more than a thousand years ago, but its active compound (artemisinin) was only extracted and verified at the end of the last century [17]. Similarly, Angelica acutiloba (Sieb. et Zucc.) Kitagawa (AAK), a herbal medicine in TCM, has been widely used for anti-inflammation [18], alleviating pain, anti-tumor [19], regulating immunity [20], and promotion of hematopoiesis function etc. [20, 21], its active ingredient still need to be elucidated, as that it contains multiple compounds, including volatile oil, phthalides, terpenes, coumarins, flavonoids, phenolic acids, and polysaccharide etc. [22]. Previous studies pointed out AAK has the function of protecting dermal tissue damage [23], inhibiting photoaging induced factors and antioxidant activity [24] of UVA-induced photoaging. Surprisingly, the function of AAK and its extracts are proved to decrease inflammatory factors, skin interstitium tissue damage and cell apoptosis etc. However, the underlying molecular mechanisms, targets and the pharmaco-active compounds of AAK for skin aging are unexplored.

The development of new drugs will last for a decade or longer time, but it is the main procession of screening ingredients with potential pharmacological effective [9]. Skin aging is a long-term progression governed by internal or external factors interacting with each other, which causes great difficulty in finding efficient active compounds. The introduce of algorithm strategy relies on network distribution, proximity and multimodal ensemble grounded on human genome-wide interaction network [25]. A drug for a particular disease might have pharmacodynamic effects for other diseases, which will take a longer time to explore. Take berberine as example, the hypoglycemic effect of oral berberine is mainly involved in decreased biotransformation of glycodeoxycholic acid, but its repurposing efficacy of berberine on gut microbiome still lack explore until the emerge of corresponding technologies [26]. Thus, new strategy should be developed to shorten the cycle of new drug development for specific diseases, aiming to explore the effectiveness and overall mechanism of action of natural medicines.

In the present study, we intend to explore the pharmaco-active components of AAK with repurposed opportunity for skin aging and their pharmacological mechanism via the proximity of NMF and cMAP connecting analysis. Subsequently the targets and mechanism of these pharmaco-active components were verified by clinical data verification from Gene Expression Omnibus (GEO) database and molecular docking. The results aimed to provide reference for skin aging in clinical practice, a theoretical strategy for identifying repurposing drugs, and new sight for our future research.

## RESULTS

### The similarity and congregate feature of AAK compound

**Figure 1:**
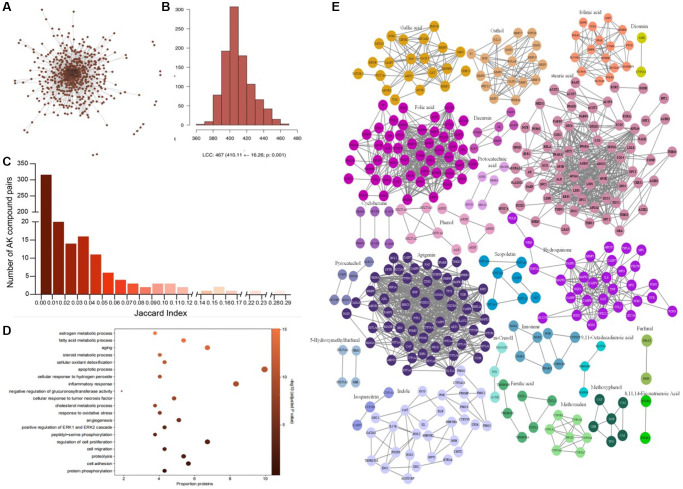
**The similarity and congregate feature of the compounds of AAK.** (**A**) Proteins targeted by AAK compounds were not randomly congregated in feature-specific adjacent region of human protein-protein interactome; (**B**) Proteins targeted by AAK formed a large connected component (LCC) consisting of 467 proteins, and multiple small subgraphs in the human interactome; (**C**) The results of Jaccard index (JI) among 29 AAK compounds; (**D**) Top (*n* = 20) enriched gene ontology terms (Biological Process)among all AAK compounds’ targets; (**E**) Protein–protein interactions of 29 AAK compounds’ targets).

Human PPI interactomes including 25120 unique proteins and 751939 pairs of interaction were collected from 20 protein-protein interaction (PPI) databases, (Supplementary Table 1). A total of 29 AAK compounds were selected after standardization and duplication, but only a few AAK compounds had a larger number of targets (Figure 1E) (Supplementary Table 2). The Jaccard index (JI) indicated that the pairs of targets among AAK compounds had a limited similarity (average JI = 0.0115) (Supplementary Table 3), which may due to the commonality of chemicals binding domains in the three-dimensional structure of protein targets [6] (Figure 1C). Gene ontology enrichment analysis recovered protein targets of AAK compounds were enriched in the same gene ontology categories and biological process. It indicated that AAK compounds target different processes of the same biological process to exert pharmacological effect as the low similarity between target proteins of AAK compound (Figure 1D). When map the targets of AAK to human PPI, most targets clustered in special region, prompting us to explore whether the interactome regions targeted by the AAK compounds reside within network neighborhoods associated with skin aging (Figure 1A, 1B).

### AAK had potential pharmacological effect for skin aging

**Figure 2:**
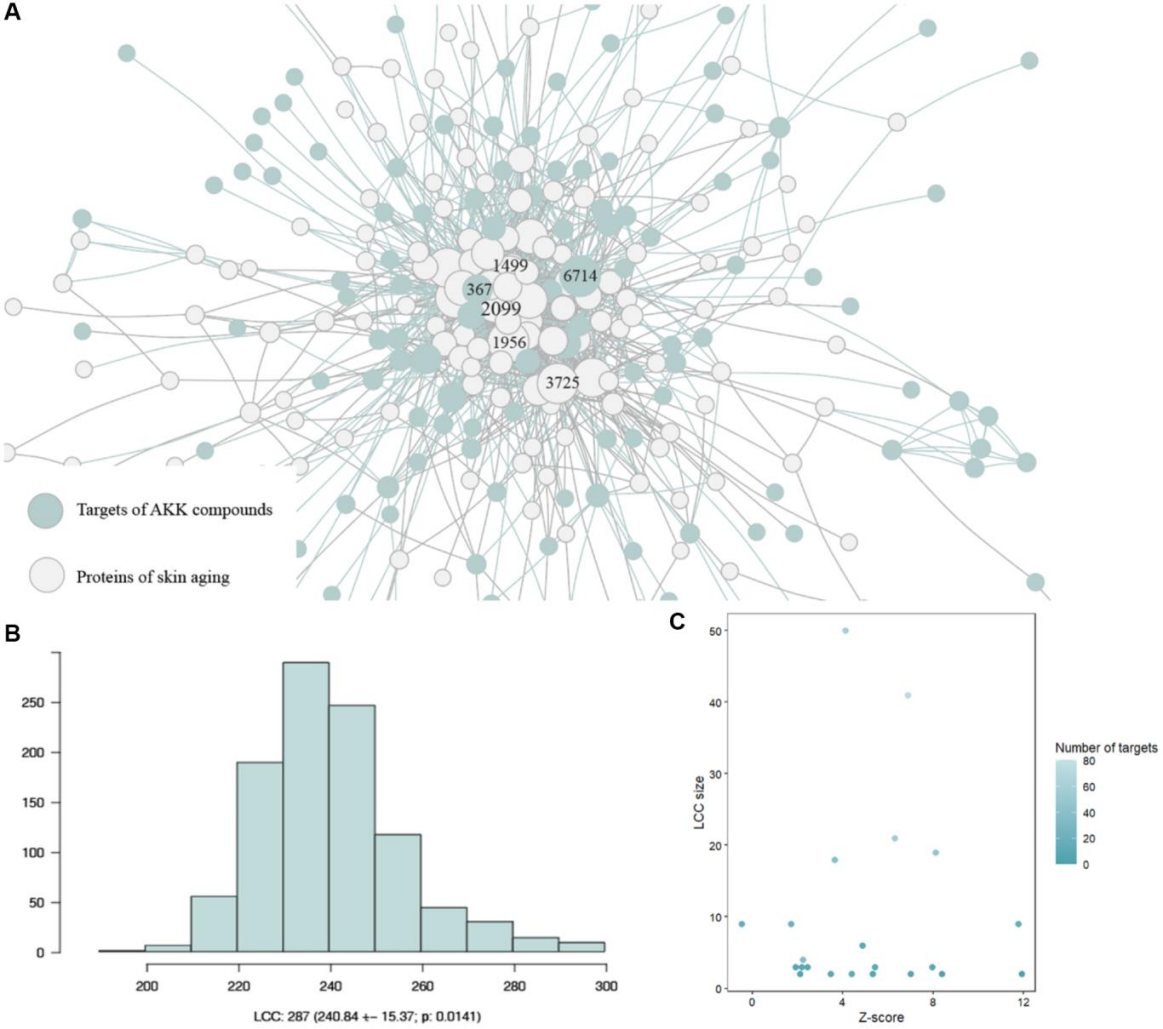
**The proteins of the AAK compounds were congregated in the feature-specific adjacent region of skin aging proteins interactome.** (**A**) AAK compound proteins congregated in feature-specific adjacent region of skin aging proteins interactome; (**B**) The random expectation of the LCC size indicates that the observed skin aging LCC; (**C**) Size of the LCC formed by the targets of each AAK compound in the skin aging protein interactome and the corresponding significance (|Z-score|).

When mapping the targets of AAK compounds to PPI of skin aging proteins (Supplementary Table 4), it showed that the targets of AAK compound were congregated in feature-specific adjacent region of skin aging proteins in the interaction network, indicating AAK compounds may cluster in the specific regions of skin aging with specific pharmacological effect (Figure 2A, 2B). We further focused on AAK compounds with more than two targets, then we measured the size and significance of the largest connected component (LCC) formed by each AAK compounds. The results indicated 23 of 29 compounds have larger LCC than expected (|Z-score| > 1.70) (Figure 2C) (Supplementary Table 5), suggesting the targets of AAK compounds may have potential pharmacological effect on skin aging.

### Proximity revealed 8 AAK compounds had therapeutic effect on skin aging

**Figure 3:**
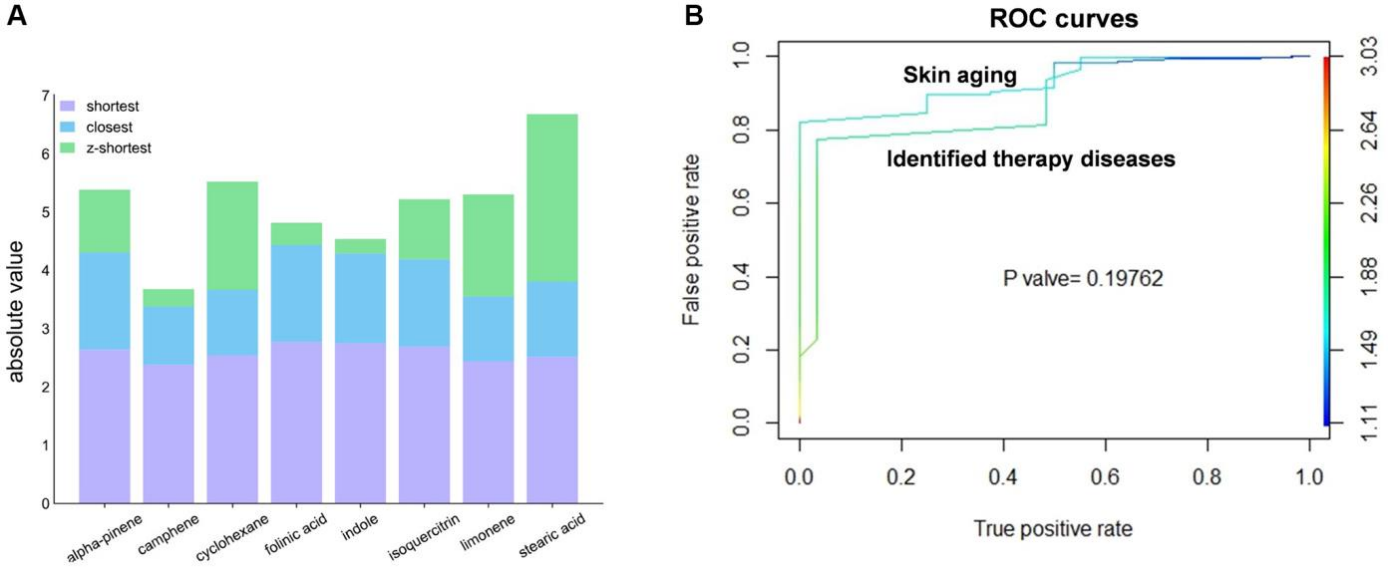
**The 8 AAK compounds had potential pharmacologic efficacy for skin aging.** (**A**) The shortest, closest and |z-shortest| calculated between compounds and skin aging by proximity and LCC analysis; (**B**) ROC curve of the 8 AAK compounds to skin aging and therapeutic diseases).

AAK compounds can be viewed as drugs in that they could exert their therapeutic effect by binding to specific proteins of skin aging, which was presented as shorter distance in the network framework. We therefore calculated the network proximity between AAK compound targets and skin aging proteins. We ranked and screened the top 8 AAK compounds with closest distance in the NMF (Figure 3A) (Supplementary Table 6), which indicated these 8 AAK compounds had pharmacological effect on skin aging, for the reason that they had shortest distance of their target cluster in the PPI network of the proteins of skin aging.

Then in the Comparative Toxicogenomic Database (CTD) database, we collected 74 diseases which can be targeted by 8 AAK compounds with known therapeutic efficacy. The proximity values of the 8 AAK compounds between skin aging and the 74 validated diseases were compared by ROC analysis, (Supplementary Table 7), and the results indicated AUC and performance accuracy were both greater than 0.75, which was similar as the AUC of AAK compounds with 74 validated diseases (AUC = 0.876) (*P* = 0.19762 > 0.05) (Figure 3B), all above results suggested that 8 AAK compounds may had therapeutic effect on skin aging.

### Medicinal effect of AAK key compounds in the treatment of skin aging

**Figure 4:**
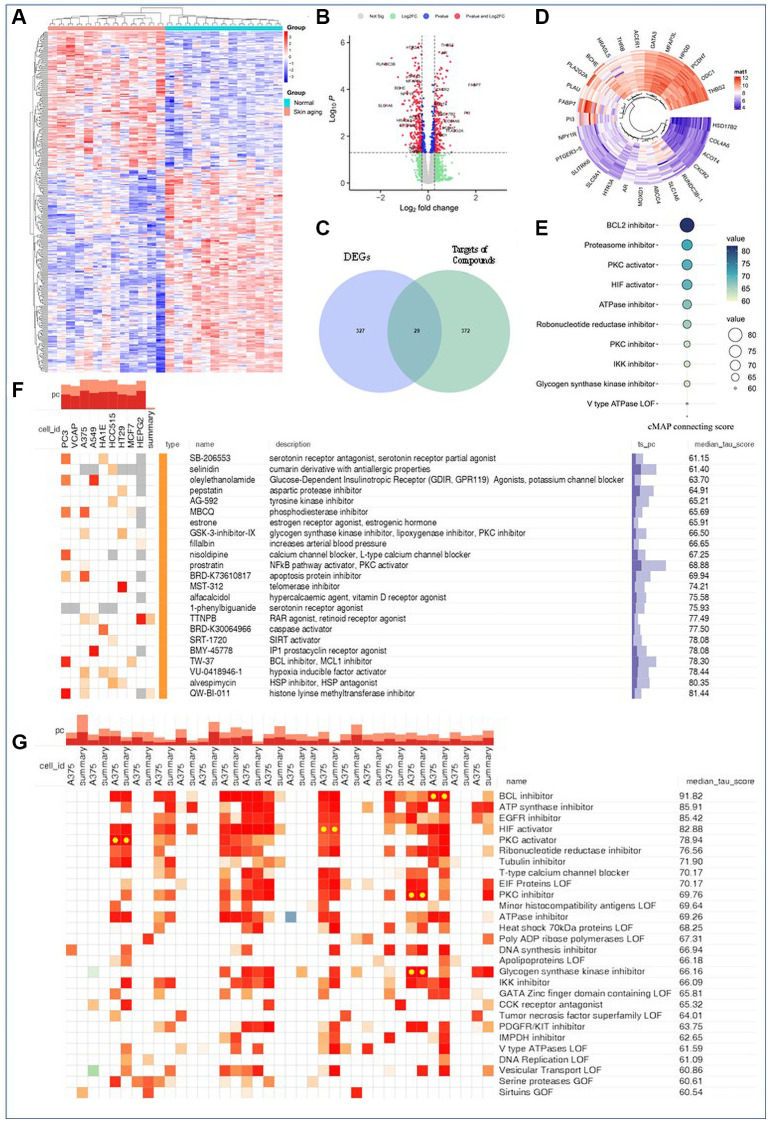
**Exploration on the function of key AAK compounds via GEO analysis and cMAP analysis.** (**A**) Heatmaps of DEGs between normal skin and actinic lentigines skin; (**B**) Volcano plot of DEGs between normal skin and actinic lentigines skin; (**C**) Venn analysis on the intersection targets between DEGs and targets of key AAK compounds; (**D**) Circular cluster heatmap of intersection targets; (**E**) Identifying compounds with similar pharmacological effect as key AAK compounds; (**F**, **G**) Identifying perturbational class of key AAK compounds and which in skin cells).

In order to further identify the potential efficacy of these key compounds, we conducted genetic perturbations analysis via cMAP database. Firstly, we identified 356 differential genes (adjust *P* < 0.05, |LogFC| > 0.5) (fold changes, FCs) from dataset GSE192564 in the GEO database, including 144 up-regulated and 212 down-regulated genes (Figure 4A, 4B). Venn analysis screened 29 intersecting genes between the differentially expressed genes (DEGs) and targets of AAK compounds (Figure 4C), including 13 upregulated genes and 16 downregulated genes (Figure 4D). As showed in Figure 4E, it indicated the pharmacological effect of 8 key AAK compounds were analogous to QW-BI-011, Alvespimycin, VU-0418946-1, TW-37, BMY-45778, SRT-1720, BRD-K30064966, TTNPB, 1-phenylbiguanide and alfacalcidol (TOP 10 of cMAP connecting score, Figure 4F). It is reported QW-BI-011 and Alvespimycin exhibit an important role in the process of skin aging. QW-BI-011 regulates epigenetic modulatory and suppresses the expression of histone methyltransferase, G9a, which restrains the development of melanoma and prevents photoaging injury [27]. Alvespimycin is a HSP90 inhibitor, it stabilizes the proteins of fibroblasts by preventing HSP90 protein upregulated [28]. VU-0418946-1, TW-37, BMY-45778, SRT-1720, BRD-K30064966, TTNPB, 1-phenylbiguanide and alfacalcidol are also intimately involved in regulating the process of skin aging. They were mainly similar to BCL inhibitor, ATP synthase inhibitor, EGFR inhibitor, HIF activator, PKC activator, ribonucleotide reductase inhibitor, Tubulin inhibitor, T-type calcium channel blocker, EIF Proteins LOF and PKC inhibitor (Figure 4G), prompting that those AAK compounds would act by managing metastasis, growth and apoptosis of cells [29–32], restraining oxidative stress [33], modulating energy metabolism of mitochondrial function [34], modulating vasodilatation [35], alone or in combination.

### The Hub-Target between AAK key compounds and skin aging

**Figure 5:**
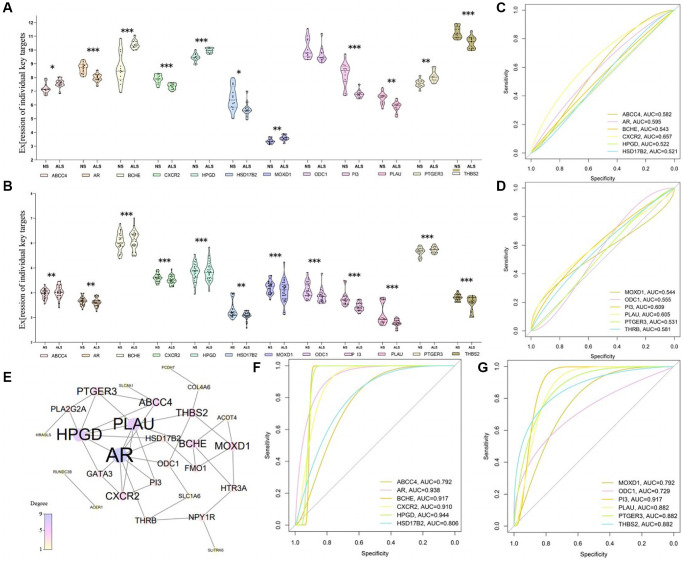
**The relationship between the Hub-target of AAK compounds and the development processes of skin aging.** (**A**, **B**) The down-regulated expression of ABCC4, PTGER3, BCEH, HPGD, MOXD1 and up-regulated expression of AR, CXCR2, HSD17B2, ODC1, PI3, PLAU and THBS2 among hub-target by AAK; (**C**, **D**) ROC curves of ABCC4, AR, BCEH, PI3, BCHE, CXCR2, HPGD, HSD17B2 through dataset GSE192564 and GSE192565; (**E**) PPI analysis of 29 core genes; (**F**, **G**) ROC curves of MOXD1, ODC1, PLAU, PTGER3, THBS2 through dataset GSE192564 and GSE192565).

Although we have obtained 29 core genes, their expression level and regulatory role during the processes of skin aging were not clarified. Thus, PPI analysis screened 12 Hub-Target from the 29 core genes according to the degree of PPI network (Figure 5E). Then, we confirmed the down-regulated expressions of ABCC4, PTGER3, BCEH, HPGD, and MOXD1 in normal group, while AR, CXCR2, HSD17B2, ODC1, PI3, PLAU and THBS2 were up-regulated in skin aging group (Figure 5A, 5B). It suggested that the key compounds of AAK played a therapeutic role by inhibiting or promoting the expression of these genes. In addition, ROC curve verified that these hub-target had certain sensitivity and specificity for the diagnosis of skin aging (AUC greater than 0.5), AR, BCEH, CXCR2, HPGD and PI3 performed well especially (Figure 5C, 5D, 5F, 5G). The AUC of them were all greater than 0.9, reminding that they could be served as specific biomarkers for skin aging diagnosis. Hence, we calculated their Cutoff value, and the results showed that when AR, HPGD and PI3 expression were up-regulated to 8.221, 9.686 and 7.603 respectively, BCEH and CXCR2 expression were down-regulated to 9.948 and 7.695 respectively, they could be applied as a criterion for diagnosing skin aging.

### The LncRNA-miRNA-target network of Hub-Target

**Figure 6:**
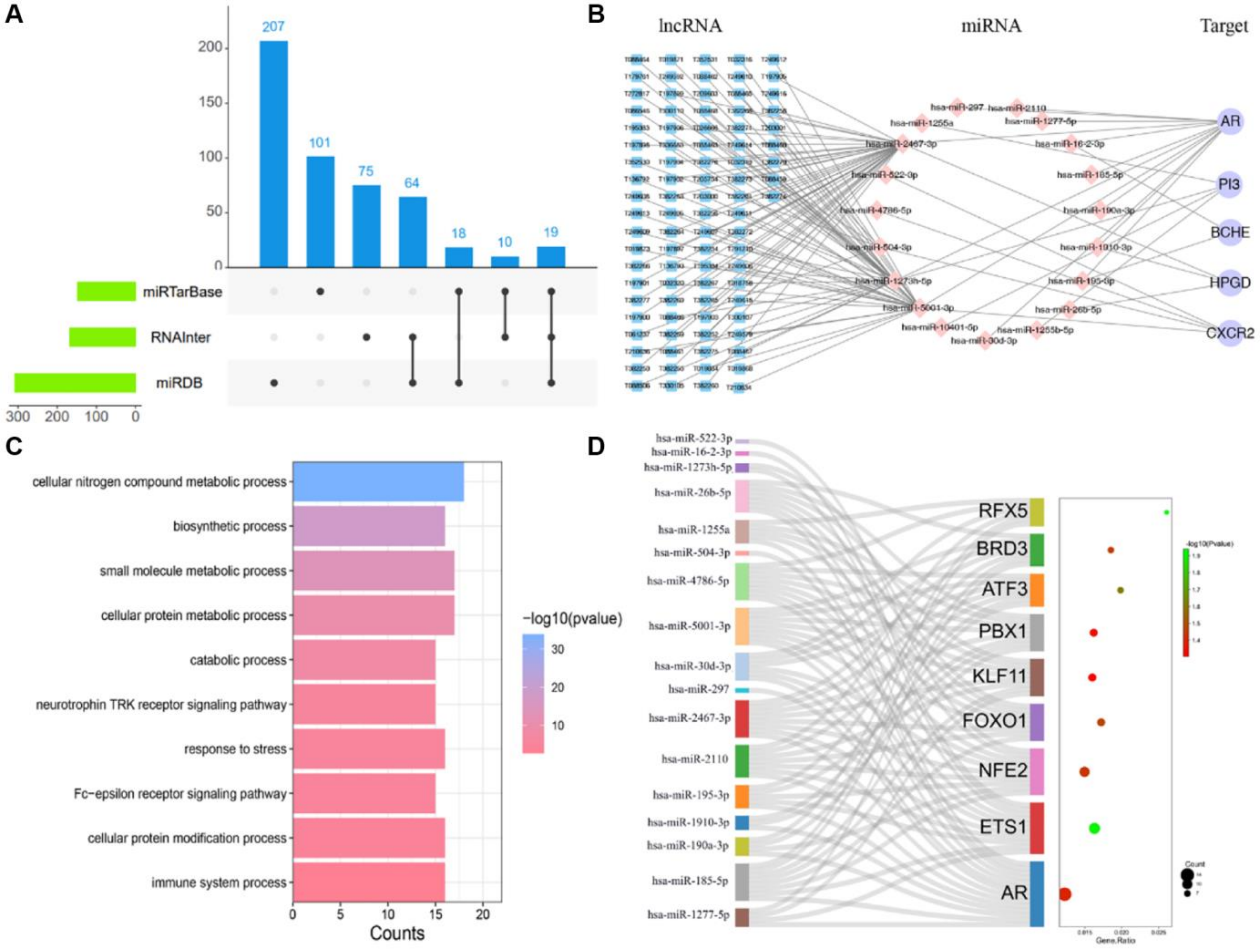
**LncRNA-miRNA-target gene network of AAK hub-targets.** (**A**) The 19 regulated hub-targets from 3 mi RNA databases; (**B**) The network of LncRNA-miRNA-target gene; (**C**) Biological processes of 19 regulated miRNA; (**D**) TF-miRNAs regulatory network).

microRNA (miRNA) is a type of non-coding single-stranded RNA molecules, it can directly join in post-transcriptional gene expression. LncRNAs are long noncoding transcripts and exposed function of target-mimetic, sponge or decoy on miRNA, their expression greatly links to specular disease and developmental stage [36]. Thus, we constructed lncRNA-miRNA interacting with AAK hub-targets to clarify the translated regulation and biogenesis pathways. As shown in Figure 6A, we screened out 19 regulatory miRNA targeting AR, BCEH, CXCR2, HPGD, and PI3 from 3 miRNA databases. They mainly participated in the biological process of cellular nitrogen compound metabolic process, small molecule metabolic process, cellular protein metabolic process, biosynthetic process, response to stress, cellular protein modification process and immune system process. The biological processes above were associated with the progression of skin aging (Figure 6C). Figure 6B manifested 90 IncRNAs (|Energy|>25 kCal/Mol, Score >150) participated in the regulatory process of miRNAs of Hub-Target. Transcription factor (TF) including ATF ETS1, NFE2, FOXO1, KLF11, PBX1, ATF3, BRD3 and RFX5 were principally involved in the regulatory process of these 19 miRNAs (Figure 6D). In brief, AAK compounds targeted skin aging by regulating these IncRNA-miRNA-target network.

### Mechanism of AAK compounds targeting skin aging

**Figure 7:**
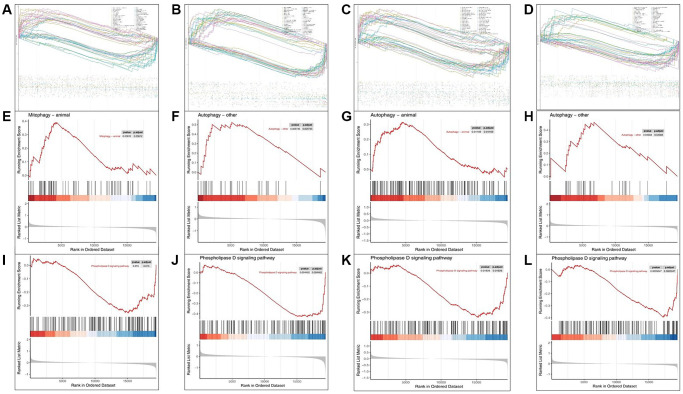
**Single-gene GSEA pathway enrichment on Hub-targets of key AAK compounds.** (**A**–**D**) Single-gene GSEA-KEGG pathway analysis in AR, BCEH, HPGD and PI3; (**E**–**H**) Single-gene GSEA-KEGG pathway enriched in autophagy pathway; (**I**–**L**) Single-gene GSEA-KEGG pathway enriched in Phospholipase D signaling pathway).

In order to explore the mechanism of AAK compounds targeting skin aging, we performed a single-gene set enrichment analysis (GSEA) on the hub targets of AAK compounds. The results showed that apart from CXCR2, other targets (AR, BCEH, HPGD and PI3) were all linked to up-regulation of autophagy (Figure 7A–7L), and down-regulation of Phospholipase D signaling pathway, which suggested AAK compounds may exert therapeutical effect on skin aging via inhibiting autophagy process and activating the Phospholipase D signaling.

### Molecular docking

**Figure 8:**
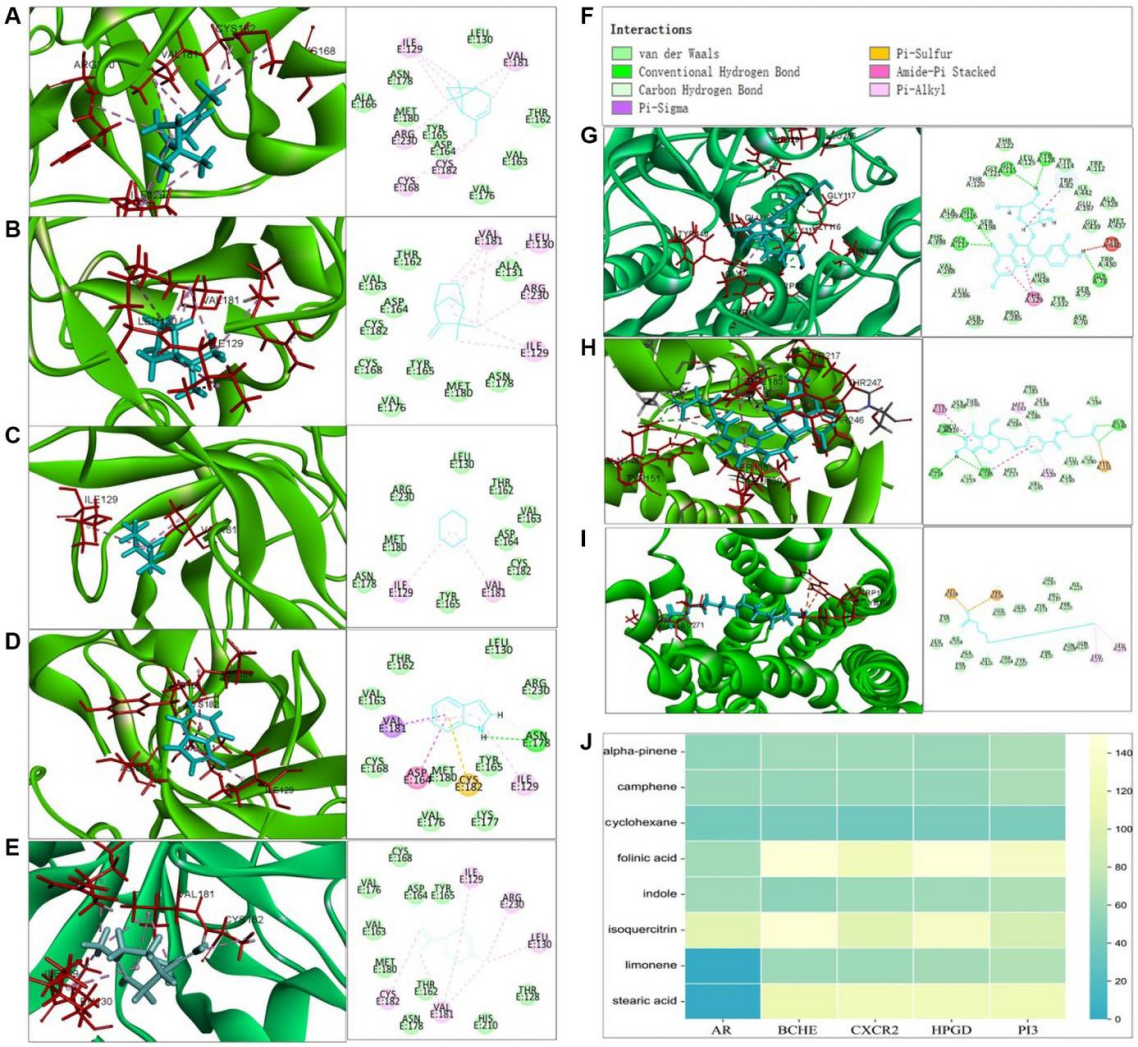
**Molecular docking analysis between AAK compounds and hub-targets.** (**A**–**E**) 2D and 3D structure visualization of alpha-pinene, camphene, cyclohexane, indole, limonene interaction docked with PI3; (**F**) type of interaction between key compounds and hub targets; (**G**) 2D and 3D structure visualization of folinic acid interaction docked with PHGD; (**H**) 2D and 3D structure visualization of isoquercitrin docked with BCHE; (**I**) 2D and 3D structure visualization of stearic acid interaction docked with CXCR2; (**J**) hot map of docking score.

The prerequisite for drug efficacy is the ability of binding with proteins or receptors. To identify whether the screened compounds could bind with core targets at protein conformations, we measured the affinity by combining approaches of spatial structure and molecular docking [37]. We calculated the docking score between the molecular structure of the AAK components (camphene, cyclohexane, folinic acid, indole, isoquercitrin, limonene, stearic acid, alpha-pinene) and Hub-Target (AR, CXCR2, BCEH, HPGD, PI3) (Figure 8A–8J), the main intermolecular forces are Van der Waals, Conventional Hydrogen Bond, Carbon Hydrogen Bond, Pi-Sigma, Pi-Sulfur, Amide-Pi Stacked, Pi-Alkyl. Indicated by docking score, the results showed that alpha-pinene, camphene, cyclohexane, indole and limonene could bind with PI3 better, folinic acid, isoquercitrin and stearic acid could bine with PHGD, BCEH, CXCR2 better respectively, indicating these 8 AAK compounds may exert pharmacological effect on skin aging by targeting PI3, HGD, BCEH and CXCR2, respectively.

## DISCUSSION

Nowadays, aging tendency of population is inevitable, aging and UVR exposure were the main drivers for the weakness of skin barrier, the decreased ability of repairing and regenerating [1]. Chinese medicinal herbs exert satisfactory role in anti-aging and has been applying for a long time, natural ingredients have become the main source for new drugs development, but it still lacks effective methods to explore effective compounds with newly pharmacology or repurposing opportunities for other disease [38].

Network medicine framework (NMF) was applied for exploring the therapeutic effects of drugs by quantifying the proximity between candidate compounds and disease and prioritizing the interacting nodes in human PPI network. The NMF plays a powerful role in quickly and correctly identifying ingredients with certain pharmacology from existing drugs, which could dramatically reduce the time and cost for novel drug development [9]. We conducted this comprehensive strategy to identify 8 active compounds (camphene, cyclohexane, folinic acid, indole, isoquercitrin, limonene, stearic acid, alpha-pinene) of AAK with drug repurposing opportunity for skin aging. Previous results showed that those compounds exhibited great effects in regulating autophagy [39], anti-inflammation [40], increasing cell viability [41], reducing antioxidant enzymes, phospholipase D [42] and lipid peroxidation [23]. Thus we made a preliminary proposal that the 8 AAK compounds would be responsible for the pharmacological effect of AAK for skin aging.

From cMAP analysis, we found that those 8 compounds had similar genetic perturbations as QW-BI-011, Alvespimycin, VU-0418946-1, TW-37, BMY-45778, SRT-1720, BRD-K30064966, TTNPB, 1-phenylbiguanide and alfacalcidol, indicating that 8 AAK compounds had same pharmacological effect as them. As reported, skin aging is involved in the process of cell proliferation, oxidative stress, apoptosis, inflammation and skin barrier repairing etc. It is reported that Alvespimycin suppressed the inflammasome/Caspase-1/GSDMD signal pathway [28], TW-37 obviously improved inflammation by inhibiting the proliferation of human oral cancer cell lines such as MC-3, HSC-3 [43, 44]. Alfacalcidol regulated inflammation process for weak elderly [45], which was similar to Alpha-pinene in down-regulating expression of inflammatory proteins. Camphene had strong antioxidant effects on scavenging the activity of hydroxyl and superoxide radicals [46], SRT-1720 suppressed the ROS generation against oxidative stress [47], TTNPB significantly up-regulated the expression of caspase-3, thus induced apoptosis of human melanoma cell [48], and Stearic acid could also induce apoptosis via same pathways [49]. Limonene and retinoids were the chemo-preventive agents for numerous or invasive nonmelanoma skin cancer, while TTNPB was a retinoid pathway activator, it had the similar pharmacological effects by acting on the same pathways [50]. Structurally, Folinic acid and TW-37 both had benzoyl group, thus they often had a similar role [51]. All above suggested 8 compounds of AAK also had similar effect on regulating these processes to alleviate the development and progression of skin aging.

Next, we further found that these 8 key AAK compounds would exert therapeutic effect by targeting AR, BCHE, CXCR2, HPGD and PI3. AR is an androgen receptor, and its expression in sun-protected skin is higher than the sun-unprotected [52], whose level is negatively correlated with the degree of damage; The activity of BCHE directly correlated with low susceptible to oxidative stress and detoxification [53]. Selective agonist to CXCR2 and PI3 kinase pathway promote human skin wound healing and keratinization, cell proliferation and migration [54], and reverse delayed skin healing as the degradation and inactivation of HPGD [55, 56]. The molecular docking proved that the high affinity between key compounds and these hub-targets, which also provided the evidence for efficacy validation.

In addition, we also found that 8 key compounds would exert anti-skin aging effect by inhibiting autophagy and activating phospholipase D (PLD) signaling pathway. Autophagy is a critical pathway against skin aging via removing aged proteins and subcellular organelles, maintaining homeostasis under external and internal stimulus such as UV irradiation and stress, and activating synthase kinase signaling pathway to protect epidermal cells [1]. Autophagy also inhibits hyperinflammatory skin reaction induced by inflammasome activation, and regulates the level of differentiated skin cells and the number of epidermal stem cells [39]. In detail, it is reported that isoquercitrin and stearic acid take part in the regulated process of autophagy by causing endoplasmic reticulum stress [49] and activating the AMPK/ mTOR/ p70S6K pathway [57]. Simultaneously, PLD and its enzymatic reaction product are the important protein in cell survival, which is regulated by the autophagosomes key component of autophagy, mTOR. mTOR combining with PLD enzymatic reaction product dual-directional regulates the formation and maintenance of the autophagosomes. The inhibitor of PLD also take part in accumulation of ceramide, which is assist in the survival of variety of cells, so that it can avoid the termination of the cell cycle [42].

The biological process of skin aging is closely related to oxidative stress, mitochondrial dysfunction, free radical accumulation and autophagy [47]. Meanwhile, this study revealed that 8 key AAK compounds had therapeutic activity on regulating oxidative stress, cellular proliferation and apoptosis and mitochondrial energy metabolism in our study. There are the reasons to believe that AAK exerts anti-skin aging effects through the mechanism of above pathways. However, due to lack of corresponding research, the regulating mechanism of 8 AAK key compounds for autophagy and PLD signaling pathway is still ambiguous, thus we initially proposed that 8 key compounds of AAK may exert anti-skin aging by regulating the autophagy and PLD signaling pathway, which drew new sights for our future research in next step.

## CONCLUSIONS

In conclusion, this study identified 8 key compounds which would be responsible for the main pharmacological effect of AAK for skin aging, these 8 key AAK compounds had therapeutic activity on regulating oxidative stress, cellular proliferation and apoptosis and mitochondrial energy metabolism in our study, and their mechanism would be involving in regulating autophagy and activating phospholipase D signaling pathway, providing a theoretical reference for identifying repurposing drugs from Chinese medicine and new insights for our future research.

## MATERIALS AND METHODS

### Human PPI interactome establishment

The human interactome was established according to 20 databases containing five different types of PPIs: (1) binary PPIs root in high throughput yeast two-hybrid (Y2H) experiments from Interactome INSIDER [58], HURI, HI-union [59], Intact [60]; (2) primary document curated from BioGRID [61], PINA [62], MINT [63], LitBM, HINT [64], HIPPIE [65], InWeb_IM [66], APID [67]; (3) low throughput kinase substrate interactions experiments from Phosphositeplus [68], KinomeNetworkX [69], Human Protein Resource Database (HPRD) [70] and affinity purification followed by mass spectrometry (AP-MS) from bioplex3.0 [71] (4) three-dimension structural analysis from Instruct [72], Interactome 3D [73], Interactome INSIDER (5) signaling interactions originated from low through-put experiments documented from Signalink. These genes were normalized according to the Entrez ID and official gene symbols through the National Center for Biotechnology Information (NCBI) database.

### Collecting compounds and targets of AAK

A total of 173 AAK compounds were retrieved from literatures and herb compounds database (PubMed, CNKI and HERB database). Then AAK compounds were screened according to the following criteria that (1) could be mapped in PubChem IDs, (2) were listed as having therapeutic effects on human diseases in the CTD database (3) had protein-binding information present in the STITCH and Drugbank database with experimental evidence. Finally, the targets of the screened compounds of AAK were retrieved from STITCH, Drugbank database, and ChEMBL database (on the threshold of homo sapiens, 90% confidence, active targets).

### Collection of skin aging disease proteins (targets)

According to CTD database, we collected the symptoms of skin aging considering the similarity level in hierarchical branches of diseases along the MeSH tree, the involved proteins of skin aging were collected from GWAS (Genome-Wide Association Studies database) and Genecards database by inputting the MESH word of skin aging or its related symptoms, including “skin aging” and “aging, skin” etc. The latter database help to clarify the human genes information like generic name, Entrez id, official symbol in genome.

### Network proximity between AAK targets and skin aging disease proteins

The proximity between skin aging and compounds of AAK were evaluated using a distance metric according to Deisy Morselli Gysi et al. [9] and Italo F. do Valle et al. [8], which takes into account the shortest path lengths between compounds targets and disease protein. The effective compounds aimed at skin aging disease proteins showed closer proximity and shorter distance. In order to evaluate the significance of proximity, we calculated the reference distance distribution and the expected distances between the proteins of compounds and diseases by randomly mapping, the mean and SD of reference distribution represented by proximity were calculated for 1000 repeats [74]. As human interactome had few nodes with high degrees due to its scale-free nature, we conducted randomly selection of nodes by stratified sampling according (binning approach) to the degree in order to avoid repeatedly selecting the same nodes with high-degree.

### AKK–disease associations

We retrieved the AAK compounds–disease associations from the CTD database, we only considered the diseases in the condition that AAK compounds have therapeutical effect, observing unconnected association at network as negative cases out of true negative cases. Network proximity was calculated based on AAK compound targets with identified disease targets from CTD database and skin aging targets. Then the AUC value of 8 AAK compounds were measured for skin aging with therapeutic and non-therapeutic diseases, meanwhile, receiver operating characteristic curve (ROC curve) was applied for comparative analysis of therapeutic targets link to compounds with skin aging. So that it could verify the predictive power of network proximity limiting the items with predictive performance of AUC >0.75.

### GEO data collection and identification of DEGs of skin aging

Expression profiling by high throughput sequencing with series number GSE192564 based on platform GPL570 (Affymetrix Human Genome U133 Plus 2.0 Array (Homo sapiens)) was downloaded from the GEO database in NCBI (https://www.ncbi.nlm.nih.gov/). The dataset contained 26 actinic lentigines skin samples and 26 normal skin samples. Then the DEGs were analyzed through the online tool GEO2R according to the |log FC| >0.5 and adjust *P* < 0.05 [75]. Hierarchical clustering and visualization were used by Heat-map plot and Volcano plot by R software (Version 4.2.0) [76]. The verification dataset (GSE192565) was also selected from GEO database and was analyzed for DEGs through online tool GEO2R according to the |log FC| >0.5 and adjust *P* < 0.05.

### cMAP analysis

cMAP is commonly applied for exploring the relationship among drugs, targets, and diseases by experimental verification result using the L1000 analysis platform, which could be used to explore or identified similar molecules sharing a same mechanism of action, chemicals and physiological processes etc. [77]. The intersection targets between key compounds of AAK and DGEs of dataset GSE192564 were imported into cMAP database to find out similar molecules sharing a same differential expressed genes to further infer the mechanism and pharmacological effect of the key compounds of AAK.

### PPI network analysis

The intersection targets between key compounds of AAK and DGEs of dataset GSE192564 were imported into the Search Tool for the Retrieval of Interacting Genes/Proteins (STRING, http://string-db.org/; version 11.0) to construct the Protein–Protein Interaction (PPI) network, then it was introduced into Cytoscape 3.1.1 software for network analysis of the core subsystem. Key targets (Hub-Target) were screened according to the degree after network topology analysis.

### Verifying the expression of key predicted targets of AAK compounds

After the hub-targets were screened, they indicated that the key compounds of AAK exerted pharmacological effect against skin aging by targeting these hub-targets. In order to validate whether these hub-targets were actually the pathogenic gene of skin aging, thus we verified the expression of hub-targets in an external cohort (the validate datasets GSE192565). The boxplot of hub-targets expression was performed using the “ggplot2” package in R software (Version 4.2).

### ROC analysis

GEO dataset 129564 (training set) and external validation datasets (GSE192565) were used to validate whether hub-targets were the specific biomarker for the diagnosis of skin aging by classifying the sensitivity and specificity of the hub-targets. The calculation of area under curve (AUC) >0.9 was seen to be of high accuracy for diagnosis, providing clinical perspective in skin aging diagnosis. The R software (Version 4.2) was used to visualize the diagnostic value of each individual gene and construct the AUC curve using “pROC” package.

### Identifying the IncRNA-miRNA-target gene network of AAK Hub-Target

miRNet database (https://www.mirnet.ca/) was used to predict lncRNA of miRNA, miRDB database (http://mirdb.org), miRWalk database (http://mirwalk.umm.uni-heidelberg.de), miRTarBase database (https://miRTarBase.cuhk.edu.cn/), and RNAInter database (http://www.rna-society.org/rnainter) were used to forecast the miRNA of mRNA (targets). Cytoscape 3.7.2 was used to construct IncRNA-miRNA-mRNA regulatory networks.

### GSEA analysis on AAK Hub-Target

To further probe the pathway involving in the mechanism of the key compounds of AAK targeting skin aging, GSEA analysis was performed on single Hub-Targets via “clusterprofiler” package (version 3.18.1) and org.Hs.eg.db package (version 3.12.0) in R software. KEGG pathways with |NES| >1, and adjust *P* < 0.05 were considered to be markedly enriched.

### Verification by molecular docking

The protein structures of hub-targets were downloaded from the PDB Database (https://www.rcsb.org/) and were preprocessed by Discovery studio software (Version 4.5). The structural files of the key compounds of AAK were downloaded from the PubChem Database (https://pubchem.ncbi.nlm.nih.gov/) and were preprocessed by Discovery studio software (Version 4.5). Finally, molecules and proteins were docked through the Libdock modular, the model with the highest docking score was selected, and their structural visualizations were conducted by Discovery studio software (Version 4.5).

### Data and materials availability

All the data could be obtained from the Supplementary Material and contacting the corresponding authors.

## Supplementary Materials

Supplementary Table 1

Supplementary Table 2

Supplementary Table 3

Supplementary Table 4

Supplementary Tables 5-6

Supplementary Table 7
