# Supplementary material for: Network medicine framework identified drug-repurposing opportunities of pharmaco-active compounds of Angelica acutiloba (Siebold & Zucc.) Kitag. for skin aging
Source: Aging (Albany NY). 2023 Jun 12;15(11):5144–63. doi: 10.18632/aging.204789 (PMC10292898; doi:10.18632/aging.204789)
Supplement: Supplementary Tables 5-6 [file aging-15-204789-s005.pdf]

SUPPLEMENTARY TABLES

Supplementary Table 5. LCC analysis of AAK target proteins on skin aging.

Compound	LCC	Z-score
Apigenin	51	3.926823
Cyclohexane	2	2.141119
Decursin	5	3.638068
Ferulic acid	3	8.156159
Folic acid	25	3.763513
Folinic acid	1	NaN
Furfural	2	3.510562
Gallic acid	8	1.245189
Hydroquinone	13	1.576066
Indole	4	1.924453
Limonene	3	2.490705
Methoxsalen	1	−0.2219654
Osthol	6	4.204503
Phenol	3	8.081199
Protocatechuic acid	2	3.854626
Scopoletin	7	20.49735
Stearic acid	42	3.554228
1-Phenyl-1,2-propanedione	1	−0.08391839
5-Hydroxymethylfurfural	2	4.96149
8,11,14-Eicosatrienoic Acid	2	31.59115
9,11-Octadecadienoic acid	1	−0.1567341
alpha-pinene	1	−0.1233418
Diosmin	1	−0.07765424
Furfuryl alcohol	1	−0.4538074
Isoquercitrin	1	−0.1427857
m-Cresol	1	−0.3505093
Methoxyphenol	1	−0.3835486
Pyrocatechol	4	2.584471
Camphene	1	NaN

Supplementary Table 6. Network proximity analysis of 8 AAK key compounds.

Disease	n_mapped_disease	n_mapped_chemical	Chemical	Shortest	Closest	z_shortest	z_closest
Skin aging	378	3	alpha-pinene	2.640212	1.666667	1.077049	1.867111
Skin aging	378	1	camphene	2.383598	1	−0.29701	−0.20752
Skin aging	378	8	cyclohexane	2.535053	1.125	−1.85766	−1.62922
Skin aging	378	6	folinic acid	2.772487	1.666667	−0.38161	0.379728
Skin aging	378	26	indole	2.749695	1.538462	−0.24965	0.259211
Skin aging	378	2	isoquercitrin	2.691799	1.5	1.029956	0.693842
Skin aging	378	9	limonene	2.439447	1.111111	−1.75109	−1.16157
Skin aging	378	73	stearic acid	2.518047	1.287671	−2.87258	−0.19628
